# Supplementary figures and images for: Structure of the helicase core of Werner helicase, a key target in microsatellite instability cancers
Source: Life Sci Alliance. 2020 Nov 16;4(1):e202000795. doi: 10.26508/lsa.202000795 (PMC7671478; doi:10.26508/lsa.202000795)

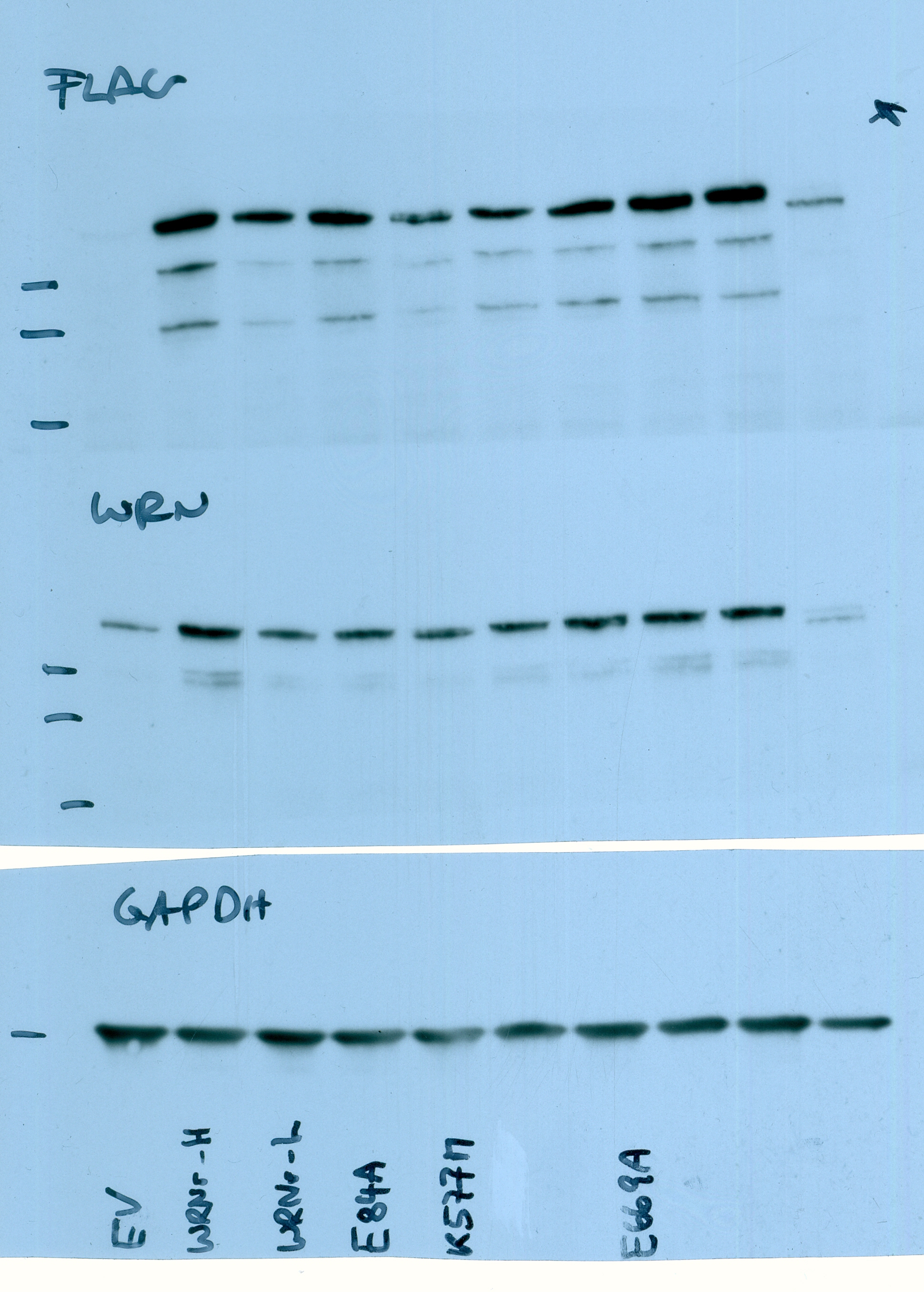

Supplement: Supplementary file 1 [file LSA-2020-00795_SdataF1.jpg]
