# Supplementary material for: Structure of the helicase core of Werner helicase, a key target in microsatellite instability cancers
Source: Life Sci Alliance. 2020 Nov 16;4(1):e202000795. doi: 10.26508/lsa.202000795 (PMC7671478; doi:10.26508/lsa.202000795)
Supplement: Supplementary file 2 [file LSA-2020-00795_TableS1.docx]

**TABLE S1. HDX Data Summary and list of experimental parameters**

| **Data Set** | WRN +AMP-PNP | WRN +ssDNA +AMP-PNP |
| --- | --- | --- |
| **States analyzed** | State 1: WRN alone; State 2: WRN + AMP-PNP | State 3: WRN alone; State 4: WRN +ssDNA +AMP-PNP |
| **HDX reaction details ^a-c^** | Final D_2_O concentration = 93.8%, pH_read_ = 7.1^a^ | |
|  | Quench buffer 1^b^ | Quench buffer 2^c^ |
| **HDX time course** | 0.167, 1, 10, 60, 240 minutes | 0.167, 1, 10, 60, 240 minutes |
| **HDX controls** | 4 undeuterated controls: 3 for state 1, 1 for state 2 | 2 undeuterated controls:  1 for state 3, 1 for state 4 |
| **Back-exchange** | 25-30% | |
| **Number of peptides** | 94 followed; 155 identified | 62 followed; 127 identified |
| **Filtering parameters** | 0.3 products per a.a., 2 consecutive products,  10ppm error | 0.3 products per a.a., 2 consecutive products,  10ppm error |
| **Sequence coverage** | 98.8% | 73.2% |
| **Average peptide length** | 13.3 | 10.7 |
| **Redundancy** | 3.00 | 2.17 |
| **Replicates** | 1 technical for each state | 1 technical for each state |
| **Repeatability** | +/- 0.15 relative Da | |
| **Significant differences** | > 0.5 Da | |

^a^ 16-fold dilution with labeling buffer [25 mM HEPES pD 7.5, 150 mM NaCl, 99.9% D_2_O]. 1:1 dilution with quench buffer 1 or 2

^b^ Quench buffer 1: 100 mM potassium phosphate, H_2_O, pH 2.1

^c^ Quench buffer 2: 4 M guanidine hydrochloride, 200 mM potassium phosphate, 200mM sodium chloride, 50mM tris(2-carboxyethyl) phosphine hydrochloride (TCEP-HCl), H_2_O, pH 2.1
